# Integrating patient perspectives in the development of a mobile health intervention to address chronic pain and heavy drinking in primary care: a qualitative study of patients in an urban, safety-net hospital setting

**DOI:** 10.1186/s13722-021-00230-0

**Published:** 2021-03-23

**Authors:** Tibor P. Palfai, Maya P. L. Kratzer, Natalia E. Morone, Judith A. Bernstein

**Affiliations:** 1 Department of Psychological and Brain Sciences, Boston University, 900 Commonwealth Ave., Boston, MA 02215 USA; 2 Department of Medicine, Section of Internal Medicine, Boston University School of Medicine, 801 Massachusetts Ave, 2nd Floor, Boston, MA 02118 USA; 3 Department of Community Health Sciences, School of Public Health, Boston University, 801 Massachusetts Ave, 4th Floor, Boston, MA 02118 USA

**Keywords:** Chronic pain, Alcohol, Heavy drinking, Self-management, Primary care

## Abstract

**Background:**

Chronic pain and heavy drinking are conditions that commonly co-occur among primary care patients. Despite the availability of behavioral interventions that target these conditions individually, engagement and adherence to treatment remain a challenge, and there have been no interventions designed to address both of these conditions together for patients presenting to primary care. This study seeks to incorporate the perspectives of patients regarding symptoms, treatment experiences, views on behavior change, and technology use to develop a tailored, integrated mobile health intervention that addresses both pain and heavy drinking among patients in primary care.

**Methods:**

Twelve participants with moderate or greater chronic pain intensity and heavy drinking were recruited from primary care clinics in a large urban safety-net hospital. One-on-one interviews were recorded and transcribed. Codes were developed from interview transcripts, followed by thematic analysis in which specific meanings were assigned to codes. Participants also completed a series of Likert-based rating scales to evaluate components of the proposed intervention to supplement qualitative interviews.

**Results:**

A number of themes were identified that had implications for intervention tailoring including: ambivalence about changing drinking, low expectations about pain treatment success, desire for contact with a designated provider, common use of smartphones but lack of familiarity with functions as a potential barrier to use, and strategies to maintain engagement and adherence. Evaluative ratings indicated that the proposed intervention content was perceived as helpful and the proposed structure, layout and design of the mobile intervention was acceptable to patients.

**Conclusions:**

Results supported the view that a mobile health intervention delivered via smartphone with electronic coaching is an acceptable method of addressing chronic pain and heavy drinking among patients in primary care. The interviews highlight the need to utilize an intervention approach that addresses motivation to change drinking, sets realistic expectations for change, provides careful attention to training/education of the use of technology components, and fosters engagement through the use of reminders, feedback, and personalized activities.

## Background

Chronic pain is one of the most common complaints for patients presenting to primary care which comprise over 50% of visits in some settings [1]. Behavioral interventions have been shown to be effective for pain management [2] but there have been few efforts to tailor approaches to foster integration into primary care systems [3] or address the persistent challenge of missed sessions and drop-out [4, 5]. Even with available pain treatments, health care providers are typically faced with co-occurring health conditions and unhealthy behaviors that complicate efforts to manage pain [1, 6, 7]. Among the most common and impactful of these is unhealthy alcohol use [8, 9], particularly heavy drinking (single occasion alcohol consumption of 4 + drinks for women and 5 + drinks for men). Heavy alcohol consumption has direct and indirect influences on pain management including increased sensitivity to pain [10], increased risk of depression and anxiety [11], and non-adherence to pain management recommendations including reduced medication adherence [12]. Chronic pain has a reciprocal influence on heavy drinking as it has shown to have negative impacts on alcohol use and alcohol treatment outcomes [13–16]. Despite the interacting influences of heavy alcohol use and chronic pain on health outcomes and on each other, no behavioral intervention has been developed to address these common comorbid conditions among primary care patients in a manner that is readily integrated into primary care, efficacious, and easily accessed and utilized by patients.

A preliminary integrated intervention was developed based on previous work on cognitive-behavioral and self-management approaches for pain [17] and alcohol use [18–21]. The purpose of this study was to gather qualitative data from participant interviews in order to further develop a tailored, integrated, technology-based intervention for primary care patients. The tailored intervention will then undergo usability and feasibility testing in an open-pilot trial before being tested in a pilot randomized controlled trial. The intervention-focused goals of this study were four-fold: (1) to collect information about how patients understand the association between pain and heavy drinking; (2) to explore the impact of past treatment experiences and expectations as potential barriers to intervention engagement; (3) to understand patient use and preferences for different types of technology; and 4) to elicit responses to intervention content and wireframes for the preliminary integrated mobile health (mhealth) intervention. We report here on themes from qualitative analyses that have direct implications for intervention content and approaches.

## Methods

### Design

In-person interviews were conducted with participants individually using a semi-structured interview guide.^1^ Review of research in the separate fields of intervention for chronic pain and intervention for heavy drinking contributed to the semi-structured interview to explore factors that might influence the acceptability and feasibility of an integrated intervention [18–20]. A panel with expertise in areas of pain management, unhealthy drinking, and cognitive behavioral therapy reviewed relevant literature, extracted key information about intervention facilitators and barriers, treatment engagement, and patients’ capacity and preferences for technology use, and developed questions and discussion probes. Open ended questions were included to obtain information about preferences and experiences beyond those identified by previous work, thus retaining some of the benefits of a naturalistic inquiry [21] while still targeting the study goal of data collection to enhance the preliminary intervention.

### Participants

Patients were invited to participate if they were 18 years of age or older, were fluent in English, engaged in primary care, experienced chronic non-cancer related pain, and reported heavy drinking. Chronic pain was defined as pain experienced for at least 3 months and of at least moderate severity in the past week. Heavy alcohol use was determined by weekly NIAAA guidelines (> 7 for women and > 14 for men) and/or a heavy drinking episode in the past month (> 3 standard drinks on one occasion for women and > 4 for men). Patients currently using pharmacological approaches to manage either pain or alcohol use were permitted in the study if medication doses were stable (i.e. same prescribed dose for at least two months). Patients were excluded if they reported a history of bipolar disorder, schizophrenia, or complicated alcohol withdrawal (i.e. delirium tremens or withdrawal seizure), had engaged in psychosocial treatment for pain or alcohol use within the past 3 months, or intended to have surgery for a pain-related condition in the subsequent six months.

### Recruitment

Recruitment and screening took place at one of multiple primary care clinics in a large, urban, safety net hospital that serves communities with a broad range of economic and technology resources. Patients whose medical records indicated experience of chronic pain were approached by a research assistant for screening either before or after their provider visit. Advertisements were placed in clinic waiting rooms as an auxiliary recruitment strategy. Screening occurred by telephone if patients recruited by advertisement contacted the study for more information. Interviews were conducted in a private office in a unit at the hospital designated for research. Enrollment of new participants was terminated when interviews reached data saturation (the point at which content was both rich in quality and thick in quantity, and no new information emerged) [22].

### Integrated intervention outline

The proposed intervention utilizes a self-regulation framework [23] to integrate evidence-based approaches for hazardous drinking and pain including Motivational Interviewing [24], cognitive-behavioral skill training [18, 19], with cognitive-behavioral and self-management approaches for chronic pain [17, 21]. The objectives of the intervention were to increase motivation and self-efficacy to change, and provide cognitive and behavioral skills to manage pain and reduce alcohol use and consequences. Intervention content was initially designed to be delivered through a series of smartphone-based video modules that would be supplemented through brief (15 min) weekly coaching delivered by a health counselor through instant messaging. Adjunct components proposed for the intervention included access to video demonstrations of pain management strategies, scheduling and activity trackers, and personalized feedback about symptoms and progress. Demonstration features of the program were created in order to elicit participant feedback on content, design, and format to later refine and tailor the intervention components, manual, and study procedures.

### Data collection

A one-on-one, 60-min interview was then conducted by researchers who had no prior association with the patient; either a research clinical psychologist (White, non-Hispanic, male with 20+ years of clinical experience) or trained research assistant (White, non-Hispanic, female with four years of interviewing experience) to elicit patient experiences and responses that might inform the intervention. To begin, the interviewer asked participants to share their experiences with pain including diagnosis, duration, interference, and pain management approaches (e.g. physical therapy, pharmacological, complementary health strategies, etc.). A brief discussion of alcohol and substance use patterns followed, including the contexts in which alcohol and substances were most often used, the role of alcohol and substances in pain management, and prior or current treatment experiences, including referrals for treatment in the primary care setting and perspectives on the need and ability to change alcohol use. The interviewer then asked about technology use and access, with particular attention to participants’ familiarity with the use of apps, instant and text messaging, and videoconferencing technologies. A broad overview of the proposed mhealth intervention was provided and participants were asked for their initial impressions before reviewing key content areas for the proposed modules. Participants rated the content areas and tools in terms of their perceived usefulness/helpfulness (“1” not at all – “5” very) and provided open-ended feedback.

In the final section, patients viewed sixteen wireframes on an iPad characterizing different intervention components and offered their general impressions. Wireframes included static images of the following intervention components and designs: weekly modules list, pain management skills demonstrations, pain, activity, and alcohol use trackers, pain management strategy scheduler, health coach appointment scheduler and instant messaging, and examples of push notifications launched from the program. See Table 1 for a detailed description of each wireframe and intervention component.

**Table 1:** Description of Wireframes

Wireframe Slide	Intervention Component	Description
Slides 1 & 2	Layout/home screen	Provided a brief description of the program’s purpose and instructions for accessing the menu. Menu content included “modules, tracker, progress, scheduler, strategies, and coaching”
Slides 3 & 4	Modules page	Provided an overview of the different topics for each week’s 10-min lesson and a sample of module 1 content: understanding how pain is related to alcohol use and medical conditions
Slide 5	Video skill demonstration	Featured an example of how pain management strategies would be introduced through video demonstrations, in this case, for progressive muscle relaxation
Slide 6	Tracker	Presented an overview of the tracker feature which would allow participants to track pain intensity, triggers, and coping mechanisms for a given day
Slide 7	Progress	Displayed how progress would be tracked and graphed weekly for both alcohol use and pain intensity
Slide 8	Feedback	Provided information and personalized feedback regarding participant alcohol use in comparison to national averages
Slides 9 & 10	Strategy scheduler	Provided an overview of how to use the program scheduler to track goals and set up specific times and reminders to practice strategies each week
Slide 11	Post-strategy ratings	Presented how to evaluate mood and track success after completing a strategy or activity
Slide 12	Strategies tab	Displayed where to quickly access the compiled list of strategies for a quick reminder
Slides 13 & 14	Coaching	Demonstrated how to schedule appointments and access the health coach via instant messaging for personalized participation
Slides 15 & 16	Reminders	Provided examples of four types of push notifications the participant would receive in the program: reminders to use the tracker, to practice sessions, to attend an appointment with the health coach, and to start newly added modules
Slide 17	Sample module	The last slide included a sample module video which gave an overview of the program as an example of the program’s weekly modules

Participants then watched a brief video of a sample module which provided an overview of the program. This section included probes about the format, layout, and usability of each intervention component, with particular attention to options for engaging with the health coach, and methods of increasing participation and engagement. Lastly, participants were asked to provide suggestions about how to improve the intervention based on what would be most helpful to them personally.

### Analytic measures

Interviews were recorded and transcribed for qualitative analysis. Transcriptions were uploaded into NVivo® v12 software for analysis. Inductive codes were developed for information that was specifically requested (questions and prompts). Deductive codes were assigned through line by line reading and discussion of content until the point of consensus. Two study investigators (TP, JB) and an external consultant participated in the process, which began with developing initial first and second level codes independently, and then meeting to compare and refine them, using principles of code definition and differentiation [25], until agreement was reached and reconciled codes were entered into the software codebook. These final codes constituted the dictionary that was applied to all transcripts. Thematic analysis of the data followed, in which specific meanings were assigned by the team to code content retrieved from NVivo. In a final stage, themes were sorted according to their potential for tailoring specific intervention components, with results presented to the entire study team for refinement. One participant declined to be recorded for the qualitative interview, and thus only quantitative ratings for this participant are reported. Descriptive analyses of Likert format questions about preferences for types of content were performed using SPSS v. 24.

## Results

### Sample Characteristics

There were 12 interviews conducted over a span of five months in 2019. Mean interview length was 55 min (SD = 14 min). Of the 12 interview participants, 7 were female, 10 were Black or African American, and 3 identified ethnicity as Hispanic. Mean age was 52.7 (SD = 10.0). Patients experienced moderate to very severe chronic pain ranging from 4 to 9 and a mean pain severity rating of 7.17 (SD = 1.2). Participants reported a mean of 18.8 drinks per week (SD = 15.0) and a mean of 8.7 (SD = 6.0) heavy drinking episodes in the past month.

### Themes from qualitative analyses

Themes from qualitative analyses with direct implications for the intervention were organized into six categories: (1) participants’ past experiences with treatment for pain or substance use, (2) reasons for drinking and (3) potential motives to change drinking, (4) technology use and (5) barriers to technology use, (5a) lack of familiarity and fear of new technology, (5b) the importance of privacy and trust in a mhealth intervention, (6) ways to foster intervention engagement, (6a) through support, results, and positive reinforcement, and (6b) participants’ need for autonomy and choice. Selected quotes for each theme are provided in the text below; additional illustrative participant statements and subthemes are presented in Table 2.

**Table 2:** Intervention themes, subthemes, and supplemental quotes

Intervention themes	Subthemes	Quote
1. Experiences and expectations of treatment	Intractable pain	*“Long. Years. Of misery. And still no results.” [Participant 12, Black male in his 50′s]*
	Helpless re: pain	*“But I mean, when it’s not the weather it’s still, you know it’s still there. The showers and the baths not gonna do anything.” [Participant 6, Black female in her 50′s]*
	Treatment history and temporary results	*“I’m just tired of taking pills and it’s not working. You know what I’m saying? It’s like okay I sit here, I don’t do nothing. I take the pills and it you know, it sustains some and so it gives me a little bit of motivation but like still. It’s just constant pain. It’s just constant pain. It gets aggravating.” [Participant 12, Black male in his 50′s]*
	Barriers to care	*“Well, I uh plan to go to acupuncture very soon. […] So I hope I’m one of the people they can actually get in because it’s on a first come first serve basis. Get there early and you know, get some acupuncture.” [Participant 5, Black female in her 50′s]*
	Treatment expectations	*“A lot of people want the instant results. And if they don’t see the instant results, ‘pfft, what I’m I doing it for?’ […] The individual, the individual themselves has to realize it’s not a microwave. Everything can’t be done quick.” [Participant 3, Black male in his 30′s]*
2. Reasons for drinking	Enjoyment/relaxation	*“I feel comfortable with it [current amount of drinking]. Like it just gets me where I want to be. I mean I don’t want to get drunk drunk. I just want to be mellow and then after that go to bed.” [Participant 1, White Hispanic male in his 60′s]*
	Social interaction	*“it’s more of a social thing as opposed to an addiction or becoming a problem for me.” [Participant 10, Black female in her 40′s]*
	Routine	*“I just go home, I drink, and then I go to bed.” [Participant 4, Black female in her 60′s]*
	Sleep	*“And sometimes for sleep I know I would drink alcohol to help me sleep you know cause the pain pills is hard to get. Especially these days […] So right now my pain is probably worse when I’m trying to go to sleep so if I tend to drink alcohol, that’s probably why. Cause it helps me go to sleep.” [Participant 7, Black male in his 50′s]*
	Mood	*“I was in a lot of pain, like as far as physically and emotionally, you know. Like I lost a sibling so it was you know, I would say maybe I was kind of self-medicating with like alcohol. Cause at that point I used to drink to the point where I would pass out. So that I wouldn’t have to feel anything.” [Participant 11, Black Hispanic female in her 30′s]*
	Distraction	*“I’m thinking about stopping the drinking, going through the program, stop the smoking you know? But I got to do- my limbs gotta be good so I could try and do other stuff to occupy that time. Cause all of that is part of that addictive behavior, you know what I’m saying? I got to substitute it with something, you know what I’m saying? I got to be able to walk in the park, do something just, you know what I’m saying? When you get that urge or that crave, you know what I’m saying? You can occupy the mind with something else, you know what I’m saying?” [Participant 12, Black male in his 50′s]*
	Numbing	*“I was just drinking to block everything. It’s pretty much a self-medication. I get a numbness from it. Like you’re not there, the pain is not really there.” [Participant 4, Black female in her 60′s]*
3. Motives to change drinking	Low readiness to change	*“I haven’t quit where it was full me. Where I made the decision. It’s been either the decision was made for me, as in um, a ruined relationship where I felt like if I quit drinking because the relationship ended I could be better in the next relationship. And I quit for… I quit for my ex more than I quit for me.” [Participant 3, Black male in his 30′s]*
	Reasons to change	*“ And I’m trying not to drink that much cause you know, some people use alcohol to suppress their depression. So I don’t want to fall in that category. You know, bad enough I can barely walk and imagine barely walking and walking around drunk or… you’re an accident waiting to happen. I got to take care of myself at the end of the day. You know? Not too much else I can do.” [Participant 12, Black male in his 50′s]*
	Barriers to change	*“Not really, I know the dangers of it and I know I probably shouldn’t but right now it’s what works for me.” [Participant 7, Black male in his 50′s]*
4. Use and perceived value of technology	Limited computer access	*“No, I have to go to somebody to get on one [a computer].” [Participant 2, Black male in his 60′s]*
	Daily smartphone use	*“I use at least 4 apps, at least 3 times a day.” [Participant 5, Black female in her 50′s*]
	Videochatting/messaging	*“I do like it. Especially now that my grandson- I have two grandkids, so they Facetime me every day so, I love it.” [Participant 8, White Hispanic female in her 50′s]*
5. Barriers to a smartphone-based intervention		
5a. Lack of familiarity with and fear of new technology are key barriers to smartphone intervention	Unfamiliar and disorienting	*“No, my friends does that. I don’t- you gotta download a plan or something in your phone they told me. Why people gotta see you? While I’m talking to them?” [Participant 12, Black male in his 50′s]*
	Openness to learning	*“No, cause I don’t know how to do that yet [watch videos on phone]. And I wish I did.” [Participant 6, Black female in her 50′s]*
5b. Critical need for privacy, trust, and some limited contact with a provider	Security, privacy, legitimacy	*“I don’t think I trust it. I would like the human opinion. Not this kind- I don’t go for that. Just as a whole- I, I don’t trust that.” [Participant 1, White Hispanic male in his 60′s]*
	Desire for in-person contact	*“But like I said every now and then I want to sit down and just talk. Face-to-face.” [Participant 2, Black male in his 60′s]*
6. Ways to increase engagement and adherence		
6a. Support, results, and positive reinforcement	From coach	*“I think it would be very helpful. I mean, you know to help you reach your goal, and you know, like just knowing that someone was helping you to reach the goal. Whether it’s managing pain or not drinking, you know.” [Participant 5, Black female in her 50′s]*
	From content of intervention	*“I think having a set time, you know? And like a, a certain number of days that you actually do it could be helpful because sometimes I just don’t feel like doing things but if it’s something that I really should do, then I just push myself to do it.” [Participant 5, Black female in her 50′s]*
	From rewards	*“If they offered something that’s real. Not just giving you a gold star, you know? I think that’ll make people do even more.” [Participant 1, White Hispanic male in his 60′s]*
	From incentives	*“Incentives inspire, inspire everybody […] When you’re setting your goal, give yourself a goal but give yourself an incentive to get to that goal.” [Participant 3, Black male in his 30′s]*
6b. Importance of autonomy and choice	Value of having choices	*“I think all that you have to offer [in regards to health coach interaction options]. The texting, or even a phone call. Yeah. Cause some people might not want to just talk.” [Participant 10, Black female in her 40′s]*
	Value of convenience	*“Yeah, I said that because you can- you’re home, and you ain’t gonna go nowhere, and they showing a fist [progressive muscle relaxation tutorial]. That’s something you can do easily. Anywhere in any part of the house you can do that. You know? Just out on the porch you know, just by yourself.” [Participant 7, Black male in his 50′s]*

#### Experiences and expectations of treatment

Many participants described pain as constant and disruptive for significant periods of time. Some also mentioned feeling helpless and resigned in response to their pain.

*“Yeah so I’m always, I’m always hurting. And I’ve hurted so long it almost became something that I’m just normally adapted to, I done become adapted to the pain.” [Participant 10]*

*“Nothing [relieves the pain], I just live with it.” [Participant 8]*

Participants had tried a variety of pain management strategies and treatments over the years including self-management strategies, physical therapy, acupuncture, aquatic therapy, pharmacological, and surgical intervention. Participants’ experiences with the efficacy of treatments varied but benefits were mostly temporary.

*“And I hate the fact that when I go to the hospital I basically gotta start from, I guess the ground level up. Always switching doctors- ‘You wanna go to therapy? You wanna do injections?’ I already had 15 of those.” [Participant 7]*

*“I don’t want to double up on the medication. It only says take once so once that one dose wears off, I’m back at square one and that’s when I’m going to the cream, use the ice packs, and the back exercises that you know my doctors gave to me.” [Participant 10]*

*“They helped for the moment. It’ll get me probably through say, 7 or 8 hours if you know, I go get the injection. It takes, it almost helps me to forget that hey, it’s no longer a condition anymore. But then once that pain- that medicine wears off, it’s back to where it started.” [Participant 11]*

Participants who had prior experiences receiving treatment for alcohol and/or substance use experienced similar difficulties attaining sustained change.

*“Nah, I liked everything about it [detox program]. Just what I don’t like about it is just that I constantly go back.” [Participant 12]*

*“If you try, and you see the results, but the treatment is ending and you don’t have the resources, then you fall back to what you know.” [Participant 1]*

Even when treatment options were available, participants noted barriers such as scheduling conflicts with work, location of and transportation to care, delays to follow-up appointments, and cost and limited insurance coverage, among others.

*“Yes, uh physical therapy and um- but my schedule wouldn’t work because I get my schedule once a week so it’s very hard to, to have um appointments for physical therapy when I don’t know when I’m working the next week.” [Participant 4]*

*“I can’t go in and say hey, give me some Oxycodones. They say no they’re addictive […] Uh well short of that it’s the surgery. But I don’t want that- I want the less invasive surgery with the needle, you know. And I have no control over that [no insurance coverage].” [Participant 7]*

Participants expressed that expectations of treatment efficacy would depend on how long the individual had lived with pain or substance use and recognized that change was a slow process.

*“So in general, I feel like it really depends on the person itself. If it’s somebody that’s been probably dealing with like pain long term- they’ve learned somewhat how to manage it. […] Now if it’s somebody that […] hasn’t been dealing with it for a long time, they’re gonna want a quick fix. They’re not going to be as patient. […] When you’re in pain, not a lot of people have patience. Unless you’ve been dealing with it for a while.” [Participant 11]*

#### Reasons for drinking

Participants mentioned using alcohol primarily for enjoyment and relaxation, as a social activity, and as part of a routine to provide a consistent activity each day.

Enjoyment/relaxation: “*it puts me in a happy space. And um, sometimes it’s just you know, to unwind, I guess.” [Participant 5]*

Social: *“I mean, it makes me happy. I don’t know […] I don’t know why I continue drinking it. But just to have a little good time with my friends.” [Participant 8]*

Routine: *“I’ve been drinking for so long, what am I gonna do? Sit down and twiddle my- I mean, come on you know, I mean I could go out for a walk. Come back home. Once you’re used to sitting down with a beer it’s like hard to get- hard to say no to it, you know?” [Participant 1]*

Participants additionally used alcohol to cope with difficulty sleeping, stress, and as a form of distraction from or numbing of the pain.

Sleep: *“It makes me go to sleep but it don’t do nothing for the pain, maybe- it might dumb it or I might forget about it for a little while but when I wake up stiff, hurting, moving, it’s pain, pain right back. But it’s better than not taking nothing at all.” [Participant 7]*

Mood: *“Especially when I get off work it’s very hard um, sometimes I’m limping and uh yeah. So it’s just pain. Constant. And that’s uh, a lot of that has to do with some of my drinking cause I will stop and get beer and just go home and drink it and relax.” [Participant 4]*

Distraction from pain: *“Well to me, when I do drink it takes my mind off of it. You know what I’m saying? I don’t really think about it, ya know. It’s just, I’m here, having a couple of drinks, playing some dominos, playing some spades. My mind is off it until I start walking.” [Participant 12]*

Numbing the pain: *“It numbs me up [laughs]. And it just, it just numbs me. For a while. Cause I know, after that it’s still gonna be the same way after I, you know, after you know it’s all said and done.” [Participant 6]*

#### Motives to change drinking

Participants varied in their motivation to change current drinking. Readiness to change levels were generally low, with approximately half of interviewees stating they weren’t considering changing their alcohol use. However, even those with low motivation to change were able to identify personal goals that were negatively impacted by their alcohol use. Some participants did identify concerns about their current use and mentioned barriers to change such as not feeling ready and lacking alternatives to cope with pain.

Health: *“I don’t do much when I drink so um but I have a goal just to stop completely […] because I’m getting older and I want to be healthy and um, I don’t know if it’s actually healthy to consume alcohol you know, as you begin to age.” [Participant 5]*

Work: *“I’m not gonna get up at 7 o’clock in the morning and have to be out the door at 8 o’clock and if I’ve been out all night, forget it. That ain’t gonna happen.” [Participant 7]*

Relationships: *“Cause I go a lot of times to my daughter’s school so I don’t- that why I don’t want people writing bad stuff about me or what not. So I make it a point not to [drink].” [Participant 7]*

#### Use and perceived value of technology

While some participants did have access to a computer for personal use, they were not used frequently. Only one of the twelve participants had access to a tablet.

*“I haven’t used it in years. I got one of those old big dinosaurs left.” [Participant 12]*

*“I don’t know how to use it that well yet. The only thing that I know how to get on is my Facebook. That’s terrible but that’s what I do know how to use.” [Participant 6]*

However, all participants used smartphones on a daily basis and unlimited data plans were common. Most participants used apps daily but their degree of integration into participant’s lives varied.

*“My whole life is on my phone.” [Participant 11]*

*“I’m kinda new to this technology stuff. I just started getting into it maybe a year ago.” [Participant 7]*

Use of text messaging and IM was common, though frequency of use varied significantly.

*“I’m not really into texting. My children text me. I receive more than I send.” [Participant 4]*

*“I’m constantly texting. That’s like my means of communication. I don’t really talk much on the phone, it’s mainly texting.” [Participant 11]*

Experiences with videochatting ranged from “never tried” to “use daily” and were most commonly used for communicating with family members.

*“And my mom she’s sick […] I’m able to Facetime her and see that she’s okay. So I see the benefits of having it now.” [Participant 7]*

*“Very rarely do I do videocalls and I can- like honestly I’ve only done that a few times and the last few times I’ve done it is only because my granddaughter called me. If it wasn’t her, I wouldn’t answer it. Most of the time, I don’t answer it.” [Participant 3]*

#### Barriers to a smartphone-based intervention

#####  Lack of familiarity with and fear of new technology are key barriers to smartphone intervention

Some participants who did not regularly use videochatting found it to be strange, uncomfortable, or disorienting.

*“I got a call from my aunt, last year, and when I answered it her face popped up. And I almost flew off the chair… I said ‘what the hell.’ […] And she’s just talking to me and I said ‘listen, listen. Can you hear me?’ ‘Of course I can hear you, I can see you too!’ I said ‘Yes, that’s what I don’t like, get off the air. Call me normal. I don’t want to talk to you looking at you. Just call me the way, you know what I mean? Normal people.’ … It’s spooky. […] And uh, to me I mean… it’s like- the future just hit me in the face.” [Participant 1]*

However, participants generally expressed interest in different smartphone features and an openness to learning about new technology.

*“I never really got into it [new phone] but now I see how important it is to actually get into it. Because it can do so much. […] Because I want to learn how, how to do all of that and you know get on the computer and do this and that. So they got a class coming up. So I can learn how to use it.” [Participant 2]*

*“I’m kinda new to this technology stuff. I just started getting into it maybe a year ago. My daughter kept urging me to do it and I didn’t wanna do it. Just give me a flip phone and I’m good, you know. […] Now I’d really get upset if I lose my phone..” [Participant 7]*

##### Critical need for privacy, trust, and some limited contact with a provider

Several participants raised questions and concerns about the security and privacy of a smartphone-based intervention and electronic health coaching component.

*“I like it but… is it like really private?” [Participant 4]*

*“And I could look into your eyes right now and find out if you really care or don’t. I don’t know what you’re thinking about online or over the phone. Or, or who am I talking to? They could say I am this and this and that.” [Participant 1]*

Multiple interviewees additionally suggested that the program include some kind of in-person contact in conjunction with the tech-based intervention.

*“How about I could do that [in reference to IM sessions] and maybe like once in a while, come in and talk to a live person? Like how I’m talking to you? […] Because then that makes them feel better when they’re talking to somebody live sometimes. And you know, some people can relate a lot better.” [Participant 2]*

#### Ways to increase engagement and adherence

#####  Support, results, and positive reinforcement

Themes surrounding how to increase participant engagement and adherence surfaced in three areas: from the health coach, from the intervention content itself, and through incentives and rewards.

From coach: participants value knowing that someone is there to support them and help them reach their goals.

*“And they could you know, pretty much give you a pep talk […] and that encouragement is probably gonna be the biggest thing. The encouragement and the- just knowing somebody has your back.” [Participant 3]*

From intervention content: seeing results in pain management after applying intervention content enhances motivation.

*“The reward would be to get better and hope this program works that you have here. But for me- other people may need rewards or something but I’m pretty much self-motivated.” [Participant 7]*

From incentives or rewards: participants emphasized the importance of using rewards that are personally relevant and meaningful, and generally agreed that incentives or rewards will enhance motivation to use the program. Specific suggestions for ways to increase adherence included using motivational or positive phrases, positive reinforcement upon completion of an activity, and pop-up notifications via phone as reminders to engage with the program.

*“You know, just a small reward […] if you’re not drinking, with the money saved treat yourself to a nice dinner… Yeah, personalized goal. Something that, something that’s for you. [Participant 3]*

*“Just have something on your phone that- like on your set up there, that once they do it and they go through the practice, there will be some kind of cheery thing. “Yay! You did great!” You know, “Congratulations, keep up the good work!” [Participant 12]*

#####  Importance of autonomy and choice

Participants valued having choices in when and how they engaged with the program and particularly in how they engaged with the health coach. Participants were asked for their preference between using videoconferencing, instant messaging, or standard phone call to interact with the program’s health coach and responses varied considerably.

*“I wouldn’t try to take one away cause then, you know, you’re limiting their choices. You’re taking a whole choice away. I would go with all three […] I say all three until that person can say personalize it. […] But it’s gonna vary from person to person I believe” [Participant 7]*

In line with their emphasis on autonomy, participants valued the immediacy and convenience of using a mhealth program, particularly as a beneficial alternative to standard care models which involve multiple steps to accessing care, lengthy wait times, and disruptions to their daily routines.

*“The advantage of it is um, sometimes you don’t really know where to go, you have to call, you have to set up an appointment, you have to figure out how to get there. With this, it’s with you.” [Participant 4]*

### Intervention component ratings

Participants were asked to rate the proposed content areas and tools in terms of their perceived usefulness after receiving a brief description. All content areas were rated using Likert-type items from 1 (“not at all useful/helpful”) to 5 (“very useful/helpful). These intervention ratings indicate a generally positive response to intervention components. The number of participants (n = 12) who rated a given content area as “helpful” or “very helpful” are presented in Table 3. Overall, ratings suggested that participants found each of the proposed intervention components to be useful or helpful.

**Table 3:** Intervention component ratings

Proposed content areas	Number of “helpful” or “very helpful” ratings
Learning how to drink alcohol in a way that is consistent with personal standards and life goals	8/12
Receiving personalized information about current drinking patterns	9/12
Learning ways to improve and maximize sleep	9/12
Behavioral activation [pleasant activities]	10/12
Learning alcohol-related harm reduction strategies	10/12
Learning strategies to relax	10/12
Learning about pain triggers and coping mechanisms	10/12
Stress management	10/12
Psychoeducation about pain, stress, and alcohol use	11/12
Learning ways to plan and pace activities	11/12
Learning ways to manage negative thoughts	11/12
Learning ways to continue self-management after completion of treatment	11/12

### Wireframe evaluations

Participants generally found the proposed number, duration, and frequency of modules to be acceptable and feasible. In response to the wireframe layout and design, participants emphasized the importance of having simple and large print text, information introduced in phases or “bites”, and interactive and engaging elements rather than static images.

*“I would definitely use more excitement. More… live. You want to keep the attention of your watchers.” [Participant 11]*

The sample module video was of an agreeable length and presented relevant and relatable content.

*“The narrator was good and it went straight to the point. […] And it delivered a lot of things that it has to offer. And solutions that are open to people that want it. So yeah, it was set up good.” [Participant 10]*

As the wireframes were simplified mock-ups of the proposed intervention, some participants commented that they seemed unprofessional or “cheap”.

*“I mean, overall I feel like the concept of it is good. Um, just probably the little details that I like pointed out here and there. But I think overall that it’s a good idea. It’s just all of how they design it and it’s put out there. Because that’s really what’s going to determine if it’s gonna be successful or not.” [Participant 11]*

Participants were in favor of receiving push notifications for the program that would remind them of new modules, upcoming health coach appointments, or of scheduled activities, so long as these were of a reasonable frequency.

*“I think they’re actually very good and I think that it would be very helpful to people, you know. Keep them reaching their goals.” [Participant 5]*

Lastly, participants were given the opportunity to recommend changes or suggest components to add to the proposed intervention. Multiple participants felt it would be important to include a resource list or emergency numbers to access support immediately if needed and to include an in-person class or session with the health coach. Several participants indicated that they wanted to exercise control/choice over the timing and duration of interactions. These suggestions indicate the desire for personalized and prompt communication.

## Discussion

This study sought to elicit patient’s perspectives on their chronic pain and alcohol use, treatment experiences and expectations, technology use, proposed intervention content and design in order to further develop and tailor a smartphone-based intervention with electronic coaching for patients in primary care. It presents formative qualitative work that will guide the continued development, tailoring, and evaluation of this integrated mhealth approach to addressing elevated rates of chronic pain and heavy drinking among primary care patients. Although limited by a small sample size, results provided insight into participants’ views of intervention content and structure, preferences and familiarity with technology use, potential barriers and facilitators to successful engagement with the program, and opportunities for tailoring the program to better address the specific needs of this patient population. A number of important implications for tailoring emerged from the interviews and responses to questionnaires.

Starting our interview with a discussion of participants’ experience of pain and histories of substance use provided essential background into experiences with treatment and aspirations for change. In many cases, participants reported that they had already tried a variety of strategies and treatments over the years and continued to experience pain that interfered with daily life, which led to feelings of helplessness and resignation. Results provided a number of insights on how the intervention might be developed. For example, it will be important to appreciate the challenges that patients have experienced in their efforts to find adequate treatment and strategies, and validate their perseverance in continuing to look for new ways to manage their chronic pain and substance use. This may be particularly important for combatting resignation or low motivation to change. Setting realistic expectations about what the program will address, what the program will require of participants in order to see progress, and how long these small incremental changes may take will be critical. For individuals who have not yet been exposed to self-management strategies or non-pharmacological approaches to pain management, it will be important to provide a clear and detailed rationale for many of the proposed program components [26]. This will be true particularly for the program’s focus on the relationship between alcohol use and chronic pain. While only seven of the twelve participants explicitly stated they were using alcohol to help cope with pain, most participants endorsed reasons for drinking that were either directly or indirectly affected by chronic pain (i.e. sleep, stress). Given that many participants did not recognize the impact of alcohol use on chronic pain, addressing this association through psychoeducation early on in the program will be important.

Many of the patients reported drinking to enhance experience or have some form of positive incentives in their lives [27]. Participant comments regarding a lack of alternative activities to drinking indicate a potential point of intervention. The behavioral activation components of the proposed intervention will be critical to address the influence of depressed mood on pain [17] and provide alternative activities to drinking to help patients cope with pain. Strategies to help patients increase nonsubstance related reinforcers in their lives also has direct benefits for reducing alcohol use [28–30]. Thus, the behavioral activation module may help to address factors related to alcohol use in individuals who do not have the explicit goal of changing their drinking.

Even when participants found treatment options that seemed to be helpful in managing their chronic pain, a variety of barriers limited their viability. Participants identified difficulties such as significant delays to accessing care (e.g., referrals, appointment availability), complications with transportation to and the location of treatment sites, limited recognition of comorbidities, and lack of continuity in care. These interviews provide insight into how our proposed program could be uniquely suited to address these barriers with the following features: immediately accessible information and coaching via phone available in any location with Internet access, recognition of pain in the context of other lifestyle factors and conditions (i.e., alcohol use, sleep hygiene, mental health concerns), and a centralized system with a single “provider” for the duration of the intervention. Beyond addressing the factors that could impede successful implementation, it will be critical to think about facilitating and fostering engagement as well. Receiving encouragement from the health coach, motivational notifications or reminders via the electronic platform, and identifying personally meaningful goals were all seen favorably by participants.

Smartphones proved to be the most accessible and familiar form of technology within our sample as opposed to tablets or computers. Many participants either did not have access to a computer, did not use one on a regular basis, did not have a private setting for computer use, or simply felt more comfortable using their phone. In line with research on the adoption of smartphones within older adult populations [31], all of our participants had access to and used a smartphone. They were generally familiar with texting and messaging and many considered apps to be an important component of navigating daily life, suggesting that a mobile intervention could be well-integrated into their routines. Participants had a positive reaction to learning information via video modules and despite some uneasiness around videochatting technology, there was an openness to see what it had to offer. Given the prevalence of chronic pain among older adults [32, 33], reference materials and instructions will need to be developed for individuals with less technology literacy to ensure that this smartphone-based intervention will be accessible and acceptable to participants of all backgrounds. In addition to assessing technology literacy, it will be important to address participants’ concerns with the use of unfamiliar technology modalities. This will also require adjustments to the program’s content and formatting. As has been shown in other app-based intervention studies, information will need to be concise and in accessible language in order for participants to fully engage with the program [34]. Results from the interviews overall suggest that the proposed content and intervention components can be integrated into participants’ daily lives.

Participants identified concerns about the security and privacy of a smartphone-based intervention as well as the credibility of the individual from whom they would receive coaching. Efforts to ensure confidentiality and the security of the technology will be important for fostering participant trust, particularly with an older adult population. Participants wanted to know who they were talking to, what made them qualified, and whether the information would be kept confidential by the coach. Concerns of trustworthiness are a common theme expressed by participants in mHealth application research [34] and it will be critical to address any doubts about the intervention during the first intervention session. Establishing trust may be facilitated by introducing the health coach via videoconferencing as opposed to phone call and through a warm hand-off from a research assistant or medical professional.

Patient comments about the value of an in-person component indicated a strong desire for interpersonal contact as part of this intervention. They emphasized the importance of having an encouraging and accessible health coach for support and for successful engagement in the program, in line with research on human support in conjunction with eHealth interventions [35]. However, preferences for how they interacted with the health coach varied considerably, with some participants in favor of instant messaging, standard phone conversation, videoconferencing, or a choice of all three.

Autonomy, or lack thereof, was a salient theme throughout the semi-structured interview. Participants discussed feelings of helplessness in regard to sustained pain management, noted multiple barriers to accessing treatments, and in some cases felt a lack of alternatives to using substances for coping with pain. When discussing options for engaging with the proposed program, participants valued having the autonomy to choose when and where they could use it. Additionally, participants wanted to have multiple ways of communicating with the health coach, as preferences could vary by individual or by day. As is common in mHealth research, participants valued having ways to personalize or tailor the program to meet their needs or preferences [34]. Even with predetermined intervention content, autonomy and choice should be fostered within the program (intervention features, components) wherever possible. Use of this smartphone-based intervention should be framed for participants as a way of gaining control over their health and healthcare.

Participants provided valuable feedback about the program content, wireframes, and modalities of intervention delivery with specific suggestions about ways to enhance engagement. Participants rated the proposed content areas and tools highly and generally found the proposed number, duration, and frequency of modules to be acceptable. Some participants discussed the importance of the program’s appearance and design. This will be an important consideration for development as the aesthetics of the program may influence how participants engage with and value the content of the intervention itself. Suggestions for increasing engagement included keeping components interesting and visually stimulating [ex: greeting chime upon login or motivational phrases], introducing new information at a manageable pace, communicating with participants in a personalized and prompt manner and/or providing emergency numbers to contact if the health coach was unavailable, and using push notifications to remind participants about completing modules and practicing strategies.

It is important to note that this study is limited by potential social desirability bias and a small sample size. However, the strength of qualitative studies lies in the representativeness of themes rather than the representation of populations. The goal of this study was not to provide a needs assessment but to help develop features of the intervention that may be tailored to this population. Those who participated shared experiences and ideas generously and offered observations that will be key to an effective intervention.

## Conclusions

In sum, this study provides important qualitative data to help tailor an integrated mobile health intervention for comorbid chronic pain and heavy drinking among primary care patients. In addition to better understanding participants’ perspectives on the content of the proposed intervention, these interviews point to the potential value of using a smartphone-based approach which has been shown to increase the adoption, reach, and efficacy of behavioral health interventions [36]. The next phase of intervention development will incorporate the themes and participant feedback from this study in advance of testing intervention usability and feasibility in an open pilot trial followed by preliminary testing of efficacy in a pilot randomized controlled trial.

## Data Availability

Data supporting the analyses are provided in the table in transcribed form.

## Abbreviation

mhealth: Mobile health

## Footnotes

1 Interview guide is available upon request.

## References

[1] Haibach JP, Beehler GP, Dollar KM, Finnell DS (2014). Moving toward integrated behavioral intervention for treating multimorbidity among chronic pain, depression, and substance-use disorders in primary care. Med Care.

[2] Department of Health and Human Services UG (2016). National Pain Strategy—A Comprehensive Population Health-Level Strategy for Pain.

[3] Miller-Matero LR, Dykhuis KE, Clark SM, Coleman JP, Ahmedani BK (2019). Treating pain in primary care: optimizing an integrated psychological intervention based on perspectives of psychologists, primary care providers, and patients. Fam Syst Health J Collab Fam Healthc.

[4] Turk DC, Rudy TE (1991). Neglected topics in the treatment of chronic pain patients–relapse, noncompliance, and adherence enhancement. Pain.

[5] Oosterhaven J, Wittink H, Mollema J, Kruitwagen C, Devillé W (2019). Predictors of dropout in interdisciplinary chronic pain management programmes: a systematic review. J Rehabil Med..

[6] Otis JD, Macdonald A, Dobscha SK (2006). Integration and coordination of pain management in primary care. J Clin Psychol.

[7] Uebelacker LA, Weisberg RB, Herman DS, Bailey GL, Pinkston-Camp MM, Garnaat SL (2016). Pilot randomized trial of collaborative behavioral treatment for chronic pain and depression in persons living with HIV/AIDS. AIDS Behav.

[8] Edelman EJ, Tetrault JM (2019). Unhealthy Alcohol Use in Primary Care-The Elephant in the Examination Room. JAMA Intern Med..

[9] Sterling SA, Palzes VA, Lu Y, Kline-Simon AH, Parthasarathy S, Ross T (2020). Associations between medical conditions and alcohol consumption levels in an adult primary care population. JAMA Netw Open.

[10] Sullivan LE, Saitz R, Cheng DM, Libman H, Nunes D, Samet JH (2008). The impact of alcohol use on depressive symptoms in human immunodeficiency virus-infected patients. Addict Abingdon Engl.

[11] Apkarian AV, Neugebauer V, Koob G, Edwards S, Levine JD, Ferrari L (2013). Neural mechanisms of pain and alcohol dependence. Pharmacol Biochem Behav.

[12] Berg KM, Cooperman NA, Newville H, Arnsten JH (2009). Self-Efficacy and Depression as Mediators of the Relationship between Pain and Antiretroviral Adherence. AIDS Care.

[13] Caldeiro RM, Malte CA, Calsyn DA, Baer JS, Nichol P, Kivlahan DR (2008). The association of persistent pain with out-patient addiction treatment outcomes and service utilization. Addict Abingdon Engl.

[14] Witkiewitz K, Vowles KE, McCallion E, Frohe T, Kirouac M, Maisto SA (2015). Pain as a predictor of heavy drinking and any drinking lapses in the COMBINE study and the UK Alcohol Treatment Trial. Addict Abingdon Engl.

[15] Zale EL, Maisto SA, Ditre JW (2015). Interrelations between pain and alcohol: an integrative review. Clin Psychol Rev.

[16] Ditre JW, Zale EL, LaRowe LR (2019). A reciprocal model of pain and substance use: transdiagnostic considerations, clinical implications, and future directions. Annu Rev Clin Psychol.

[17] Otis JD (2007). Managing chronic pain: a cognitive-behavioral therapy approach, therapist guide.

[18] Morgenstern J, Irwin TW, Wainberg ML, Parsons JT, Muench F, Bux DA (2007). A randomized controlled trial of goal choice interventions for alcohol use disorders among men who have sex with men. J Consult Clin Psychol.

[19] Sobell MB, Sobell LC (1993). Problem drinkers: guided self-change treatment.

[20] Du S, Yuan C, Xiao X, Chu J, Qiu Y, Qian H (2011). Self-management programs for chronic musculoskeletal pain conditions: a systematic review and meta-analysis. Patient Educ Couns.

[21] Ruehlman LS, Karoly P, Enders C (2012). A randomized controlled evaluation of an online chronic pain self management program. Pain.

[22] Fusch P, Ness L (2015). Are we there yet? Data saturation in qualitative research. Qual Rep.

[23] Karoly P, O’Donohue WT, Fisher JE (2012). Self-regulation. Cognitive behavior therapy: core principles for practice.

[24] Miller WR, Rollnick S (2013). Motivational interviewing: helping people change.

[25] Guest G, MacQueen K (2008). Handbook for team-based qualitative research.

[26] Morone NE (2019). Not just mind over matter: reviewing with patients how mindfulness relieves chronic low back pain. J Evid-Based Integr Med..

[27] Cox WM, Klinger E (1988). A motivational model of alcohol use. J Abnorm Psychol.

[28] Murphy JG, Dennhardt AA, Skidmore JR, Borsari B, Barnett NP, Colby SM (2012). A randomized controlled trial of a behavioral economic supplement to brief motivational interventions for college drinking. J Consult Clin Psychol.

[29] Cox WM, Klinger E, editors. Handbook of motivational counseling: Goal-based approaches to assessment and intervention with addiction and other problems. 2nd ed. Wiley Blackwell; 2011, 641 p.

[30] Vuchinich RE, Tucker JA (1988). Contributions from behavioral theories of choice to an analysis of alcohol abuse. J Abnorm Psychol.

[31] Pew Research Center. Tech adoption climbs among older adults. 2017. https://www.pewresearch.org/internet/2017/05/17/technology-use-among-seniors/. Accessed 16 Nov 2020

[32] Dahlhamer J, Lucas J, Zelaya C, Nahin R, Mackey S, DeBar L (2018). Prevalence of Chronic Pain and High-Impact Chronic Pain Among Adults — United States, 2016. MMWR Morb Mortal Wkly Rep.

[33] Patel KV, Guralnik JM, Dansie EJ, Turk DC (2013). Prevalence and impact of pain among older adults in the United States: findings from the 2011 National Health and Aging Trends Study. Pain.

[34] Vo V, Auroy L, Sarradon-Eck A (2019). Patients’ perceptions of mHealth apps: meta-ethnographic review of qualitative studies. JMIR MHealth UHealth..

[35] Mohr DC, Cuijpers P, Lehman K (2011). Supportive accountability: a model for providing human support to enhance adherence to eHealth interventions. J Med Internet Res.

[36] Ramsey AT, Satterfield JM, Gerke DR, Proctor EK (2019). Technology-based alcohol interventions in primary care: systematic review. J Med Internet Res.

